# Development of interactive guidance for cold exposure using a thermoregulatory model

**DOI:** 10.1080/22423982.2023.2190485

**Published:** 2023-03-19

**Authors:** Xiaojiang Xu, Timothy Rioux, Karl Friedl, Julio Gonzalez, John Castellani

**Affiliations:** a Thermal and Mountain Medicine Division, U.S. Army Research Institute of Environmental Medicine, Natick, MA, USA; b U.S. Army Research Institute of Environmental Medicine, Natick, MA, USA

**Keywords:** Cold stress, cold injury, frostbite, cold weather ensemble, manikin, decision aid, personalised medicine

## Abstract

For decades, the Wind Chill Temperature Index (WCT) and its various iterations have been used to assess the risk of frostbite on unclothed body parts. This paper presents an innovative knowledge-based Cold Weather Ensemble Decision Aid (CoWEDA) that can be used to guide the selection of the most appropriate cold weather ensemble(s) relative to anticipated mission physical activities and environmental conditions. CoWEDA consists of a validated six-cylinder thermoregulatory model, a database of clothing properties, algorithms for calculating the whole ensemble properties from individual garments and a graphical user interface. The user-friendly CoWEDA allows users to select from an inventory of clothing items to build an ensemble suitable for their needs. CoWEDA predicts the risks of both frostbite and hypothermia and ensures that a selected clothing ensemble will provide adequate protection to prevent cold injury. CoWEDA predictions provide not only estimates of frostbite risk similar to WCT tables but also hypothermia times and clothing required to prevent cold injuries. In addition, a CoWEDA model variant can predict survivability and clothing requirements during cold water immersion. Thus, CoWEDA represents a significant enhancement of the WCT-based guidance for cold weather safety and survival by providing greater individual fidelity in cold injury predictions.

## Introduction

Cold weather is a persistent danger during military operations and often creates health hazards [1]. “Cold Still Kills: Cold-Related Illnesses in Military Practice Freezing and Non-Freezing Cold Injury” was the title of a review article by Imray and Oakley that highlights the dangers of operations in cold weather [2]. Every year, ~500 of United States Armed Forces service members have had at least one medical encounter for a cold injury, with a primary diagnosis of frostbite as the most common [3]. Civilian populations, e.g. outdoor workers and winter sports participants, are also at risk [4,5]. In the summer of 2022, a hiker suffered from severe hypothermia and died when hiking on Mt Washington, NH (https://www.cbsnews.com/boston/news/white-mountains-new-hampshire-hiker-death-xi-chen-andover/, accessed on 8/5/2022). This tragedy could very likely have been avoided if he knew the potential danger and brought one or two more pieces of clothing. Situational awareness and proper selection of cold weather ensembles are the primary mitigation strategies for preventing cold injury [4].

For decades, the Wind Chill Temperature Index (WCT) has been the most common tool for communicating the risks of cold weather exposure, and various WCT iterations have been used to assess the risk of frostbite on unclothed body parts [6–10]. However, WCT provides only information on the risk of frostbite, and does not provide any information on the risk of hypothermia or countermeasures such as appropriate protective clothing. The WCT in this paper refers to the chart published by the US National Weather Service (https://www.weather.gov/safety/cold-wind-chill-chart, accessed on 3/2/2023). Another popular method is the required clothing insulation (IREQ), which is incorporated into the International Standard ISO11079 [11–14]. ISO 11079 determines the insulation of cold weather ensembles required to maintain heat balance for different sets of environmental conditions and work intensity, and provides limited guidance for the evaluation of extremity cooling.

This paper presents an innovative knowledge-based Cold Weather Ensemble Decision Aid (CoWEDA) for the prevention of cold injury. CoWEDA is a software application for the assessment of risk in cold weather operations and the selection of appropriate cold-weather ensemble(s) relative to anticipated mission physical activities and environmental conditions.

## Methods

### Performance of cold weather ensembles

CoWEDA evaluates the thermal performance of cold weather ensembles through endurance times predicted by the underlying model. Endurance times are defined as the time until core temperature and toe and finger skin temperatures drop to a low level, i.e. physiological threshold, indicating a high risk of cold injuries. These metrics focus on the safety or performance of the person in cold and quantify the protection level provided by a cold-weather ensemble to maintain safe body temperatures. The thresholds used in this model are as follows: core temperature 36°C, hand and foot temperatures 5°C and skin wettedness 0.5. The threshold of 5°C was selected as it provides a conservative value and enables rewarming to take place before frostbite (<0.5°C) is a serious concern. The skin wittedness 0.5 is the upper limit of thermal comfort [15].

### Biophysical properties of cold weather ensembles

Cold weather ensemble systems from various military organisations (60 individual garments and 34 ensemble configurations) were tested on sweating thermal manikins to collect thermal and evaporative resistances [16–18]. All manikin testing was conducted according to ASTM International standards F1291–22 and F2370–22 [19,20]. Thermal resistance was measured with environmental conditions maintained at 20°C air temperature, 50%RH, and 0.4 m·s^−1^ wind speed. Evaporative resistance was measured with environmental conditions maintained at 35°C air temperature, 40%RH, and 0.4 m·s^−1^ wind speed. The data were processed and saved in the database, including clothing resistances and images. Algorithms were developed to calculate the intrinsic thermal and evaporative resistances for full ensembles from the value of individual items and the calculated values were used as clothing inputs for the thermoregulation model [17].

In the six-cylinder thermoregulatory model (SCTM, see details in the next section), total thermal resistances consist of clothing intrinsic thermal resistances and boundary layer thermal resistances. The boundary layer thermal resistances are affected by the wind speed [21].

### Six cylinder thermoregulatory model

**Figure 1.**
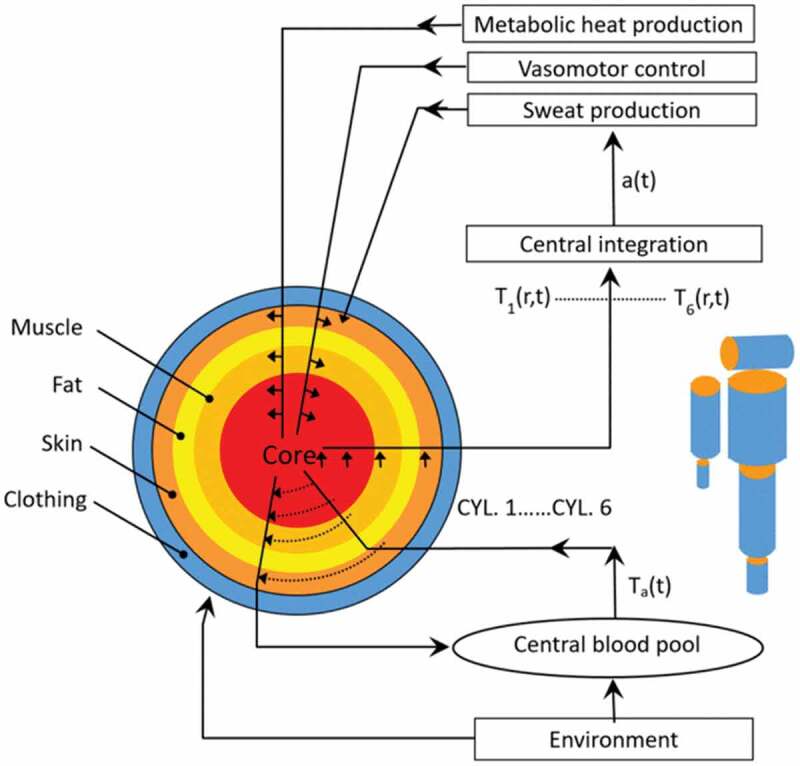
Schematic of Six Cylinder Thermoregulatory Model (SCTM).

The six-cylinder thermoregulatory model (SCTM) was used to predict physiological responses to determine endurance times. SCTM is a rational model and is based on the first principles of physiology and the physical laws of heat transfer [21,22]. As shown in Figure 1, the human body is subdivided into six segments representing the head, trunk, arms, legs, hands, and feet. Each segment is further divided into concentric compartments representing the core, muscle, fat, and skin. The surface area and volume of each cylinder were determined from the body surface area and volume using distribution factors [23]. The cylinder surface area and volume were further used to determine cylinder length and diameter. The integrated thermal signal to the thermoregulatory controller is composed of the weighted thermal input from thermal receptors at various sites distributed throughout the body. The difference between this signal and its threshold determines activation of the thermoregulatory effectors of shivering heat production, vasodilation/vasoconstriction, and sweat production.

The SCTM has been validated for a broad range of conditions (−40ºC to 48ºC), including heat, cold, and water immersion [22,24–26]. SCTM predicts physiological responses (e.g. core temperatures, skin temperatures, and sweat rates) for six body regions.

### CoWEDA — Interactive guidance

**Figure 2.**
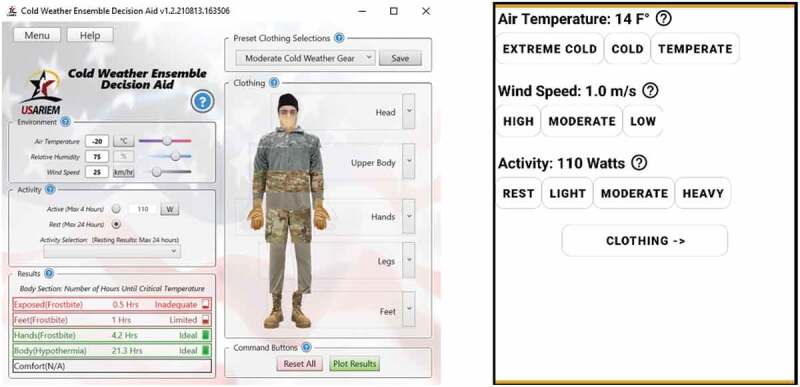
Interactive Guidance for cold injury prevention cold weather ensemble decision aid (PC version and Mobile version).

The SCTM, clothing database, and algorithms for calculating the regional thermal and evaporative resistances of a selected ensemble were integrated into a user-friendly software application, the CoWEDA [27,28], shown in Figure 2. The user-friendly CoWEDA uses a pull-down menu of military cold weather clothing to input pre-selected multi-layer cold weather ensembles or to select individual clothing items to build an ensemble. Weather inputs include air temperature, wind speed, and relative humidity. There is a menu for different activity levels, ranging from rest to heavy activity. Outputs are endurance times, which define the performance of cold weather ensembles, representing the general risk of frostbite, frostbite of hands (covered or bare), frostbite of feet (with footwear), hypothermia, and “comfort”. Comfort for CoWEDA is the upper limit for comfort and is used to avoid overdressing.

## Evaluation

CoWEDA was validated with physiological data from three studies: 1) eight volunteers sat in 0.5°C for 2 h and wore 3 layers of the Army Extended Cold Weather Clothing System (ECWCS) on torso and 2 layers on legs but were bare-handed throughout the exposures [29]; 2) six volunteers rested or exercised at two intensities in 0, −20 and −30°C environments for up to 2 h, and they wore an ECWCS ensemble and three different gloves [30]; 3) four volunteers wore the ECWCS ensemble and were exposed to −40°C environments for 2 h [31].

Comparison of the predicted skin and core temperatures with measured values during 17 cold exposures ranging from 0 to −40°C resulted in most predictions being within ±1 standard deviation (SD) of the measured mean [27,28]. In addition, differences between observed temperatures and CoWEDA predictions were evaluated using the root mean square deviation (RMSD). The RMSD for the finger, mean skin, and core temperatures were 3.30°C, 1.18°C, and 0.26°C, respectively, while the corresponding average SD of the observed values was 3.56°C, 1.22°C, and 0.31°C, respectively [28]. These CoWEDA predictions are generally acceptable for the intended applications, although hand predictions (finger temperatures) require refinement.

CoWEDA was evaluated by soldiers in Alaska during training in extreme cold. Users indicated that CoWEDA helped them decide what to wear and understand what to expect during exposure to cold. Further testing to demonstrate improved outcomes such as reduced cold injury needs to be done in the future.

## Discussion

The user-friendly CoWEDA allows users to select from an inventory to build an ensemble suitable for their environmental protection needs. CoWEDA predicts the risk of frostbite and hypothermia and ensures that a selected clothing ensemble is appropriate for expected weather conditions and activities.

### CoWEDA vs Wind Chill Temperature (WCT)

WCT has been widely used as an index for cold injury prevention. Most of the current guidance for cold stress and injury management relies on WCT, with the risk of frostbite presented as a look-up table. CoWEDA was compared to the WCT frostbite times for an individual at rest wearing the Army Combat Uniform (ACU) and a moderate cold weather ensemble (Table 1). The moderate cold weather ensemble includes a hat and balaclava, midweight cold weather shirt and drawers, fleece jacket, wind jacket, pants, and intermediate cold wet boots. The intrinsic insulation of the whole body is ~1.9 clo. Table 1 shows that the CoWEDA predicted finger frostbite times with the ACU and moderate cold ensemble are similar to those in WCT. CoWEDA predicted hypothermia times vary with clothing, while WCT does not provide any information on hypothermia risk. For the hiker incident mentioned in the introduction, the ambient temperatures were approximately 2 to −9°C according to the news, and the WCT index shows no risk at all. However, the CoWEDA prediction indeed shows the hypothermia risk only when the ACU was worn, and hypothermia may occur in 2–4 hours at −9 ºC. If the moderate cold ensemble was worn, the hypothermia risk could be reduced significantly.

**Table 1.** CoWEDA predicted frostbite time (min), hypothermia time (h) and Wind Chill Index (WCT) frostbite time (min).

		CoWEDA frostbite time (min)									CoWEDA hypothermia time (h)				
	T ºCV m/s	2	−9		−21		−32		−43		2	−9	−21	−32	−43
A	2		32		18		14		11			4.2	1.8	1.2	1.0
	11	69	15		10		8		7		22.9	2.3	1.3	0.9	0.7
	20	38	13		9		7		6		20.3	2.0	1.2	0.9	0.7
M	2		33		18		14		11				23.5	7.1	2.9
	11	93	15		11		8		7				20.1	4.5	2.4
	20	39	13		9		7		6			24	19.1	4.0	2.3
		WCT frostbite time (min)													
	2							30		10					
	11					30		10		5					
	20					30		10		5					

T: ambient temperature °C, V: wind speed m/s; A: Army Combat Uniform; M: Moderate Cold Weather Ensemble.

In addition, CoWEDA can predict the duration of effective manual performance. Impairment of manual performance is a common challenge in occupations such as fishermen and Soldiers in the Arctic. Manual performance is progressively degraded with cold exposure as hand temperature drops. Manual performance declines by 20% and 50% from normalised performance when hand temperature drops to 15°C and 5°C, respectively [32,33]. Table 2 shows that CoWEDA predicts the duration of effective manual performance, which is the time when hand temperature drops to 15°C and manual performance starts to drop rapidly. These durations of effective manual performance vary with gloves and exercise intensity. Military planners, industrial hygienists, and occupational medical specialists can use CoWEDA predictions, e.g. durations of effective manual performance, to plan operations to ensure safe and effective performance of individuals working in the cold.

**Table 2.** CoWEDA predicted duration of hand manual performance (min) when wearing the moderate cold weather ensemble.

	No glove: Rest					Glove: Rest					Glove: Exercise				
T °C V m/s	2	−9	−21	−32	−43	2	−9	−21	−32	−43	2	−9	−21	−32	−43
2	21	13	10	8	7	150	64	42	32	26			73	42	31
11	12	8	6	5	5	122	56	37	28	23			55	35	27
20	10	7	5	4	4	112	54	36	28	23			52	34	26

T: ambient temperature °C, V: wind speed m/s.

### CoWEDA vs Required Clothing Insulation (IREQ)

IREQ has been a widely used index for cold weather clothing evaluations and cold risk assessments [34,35]. The basis for IREQ is the heat balance of the whole body without any thermoregulatory mechanisms. CoWEDA is based on a validated model of human thermoregulation that considers thermoregulatory mechanisms, and local and whole-body heat balances. IREQ predicts insulation values that are required to maintain body heat balance as well as duration limited exposure and a required recovery time [12,14,34] but does not predict insulation values required to prevent extremity cold injury or local cooling. In contrast, CoWEDA predicts clothing, gloves and boots that are required to stay safe. Furthermore, IREQ was designed for professionals, and requires professional knowledge to use and to interpret the results [11]. CoWEDA was designed for end-users and is extremely user-friendly.

### CoWEDA variants

The CoWEDA architecture is designed in such a way that it can be easily adapted to create variations for different user communities (e.g. utility workers). The adaption requires two steps: 1) the physiological criteria and 2) the establishment of the clothing database. The physiological criteria reflect the operation requirements. Utility workers, for example, need to keep their hands warm to complete repair tasks in extreme cold, thus the hand criteria could be 15°C and outcome would be the duration of effective manual performance as shown in Table 2. Utility workers have their own clothing systems, and these clothing systems need to be tested to be included in CoWEDA. Then, a CoWEDA version for utility workers can be created. CoWEDA variations can help widen the scope of users to select ensembles and manage their working schedule in a rational manner.

One example of variants is CoWEDA with an immersion option, the Probability of Survival Decision Aid (PSDA). Cold water survival time is critical for certain operations in or above water. Thus, PSDA was developed by USARIEM for the U.S. Coast Guard (USCG). PSDA predicts survival time for hypothermia and dehydration during prolonged exposure in both air and water over a wide range of environmental conditions [36,37]. In June 2010, the USCG mandated the use of PSDA for all cases involving persons in the water and where persons are at risk of hypothermia.

**Figure 3.**
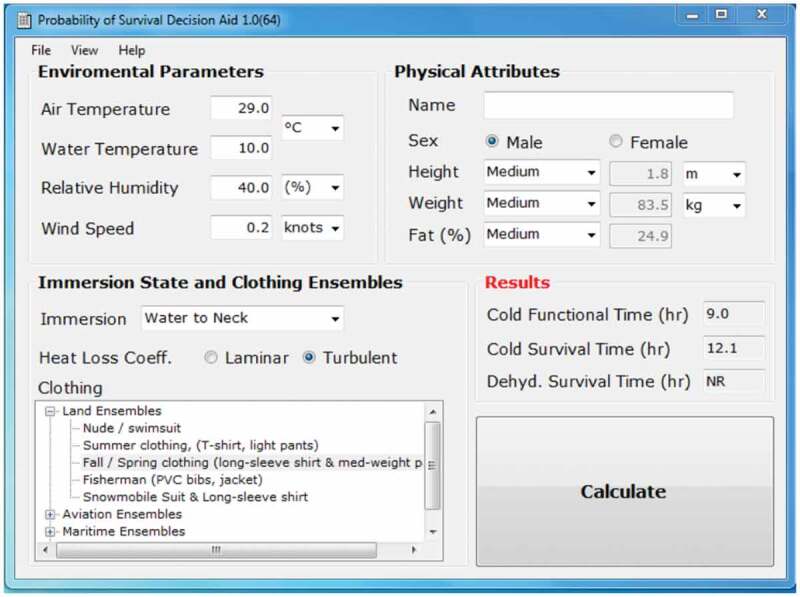
Probability of Survival Decision Aid (PSDA).

As shown in Figure 3, PSDA requires the input of individual characteristics (i.e. height, weight and %fat). PSDA was validated using reported cases for accidental water immersions. For most immersion victims for whom height and weight were known, the predicted survival time for each victim was either very close to or greater than the observed survival time [36,37].

**Figure 4.**
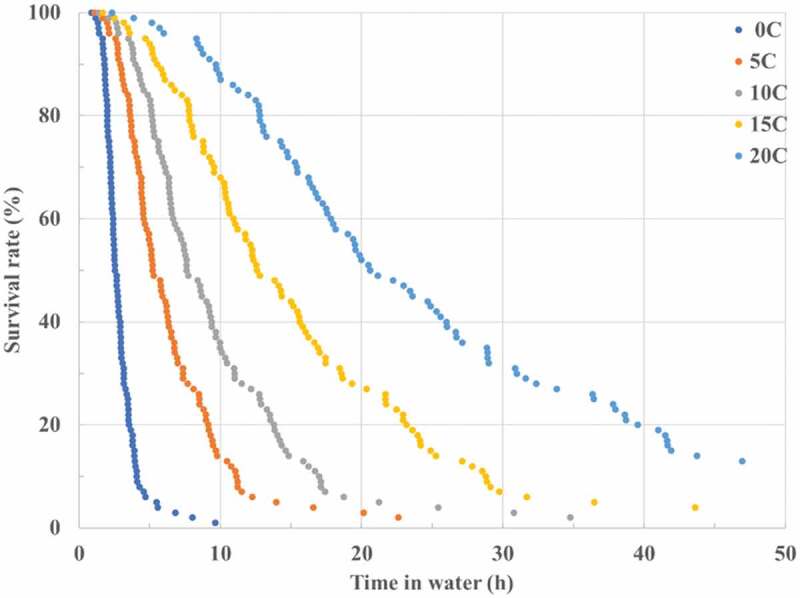
Predicted hypothermia survival time for 100 victims during immersion at 0°C, 5°C, 10°C, 15 and 20°C water.

Current survival tables in operational use provide survival times only for individuals with certain characteristics. However, differences in individual characteristics may result in varying survival times. PSDA predicted survival times of 100 victims (various height, weight and %fat) during immersion at water temperatures of 0°C, 5°C, 10°C, 15°C and 20°C. These are hypothermia survival times only without cold shock and swimming failure. As shown in Figure 4, about 50% of the test population should survive hypothermia for 2.6, 5.2, 7.2, 12.6 and 20.6 h at 0°, 5°, 10°, 15°C and 20°C water, respectively. Figure 4 also shows significant individual differences in survival time. The survival tables or curves in use are not able to provide information on this range of individual differences.

## Conclusions

CoWEDA predictions not only provide estimates of frostbite risk similar to WCT tables but also provide hypothermia times and clothing required to prevent cold injury. In addition, CoWEDA variants predict risk, survivability and clothing requirements during cold-water immersion. Therefore, CoWEDA represents a significant enhancement of the WCT-based guidance for cold weather safety and survival. Further work is needed to include other predictive factors that will refine individual prediction accuracy. These factors include cold-wet modelling, individual body composition and other metabolic factors, nutritional status, and quantitative contributions of fitness factors.

## Disclaimer

Approved for public release; distribution is unlimited. The opinions or assertions contained herein are the private views of the author(s) and are not to be construed as official or reflecting the views of the Army or the Department of Defense. Any citations of commercial organisations and trade names in this report do not constitute an official Department of the Army endorsement of approval of the products or services of these organisations.
